# Supplementary figures and images for: Dynamic changes of Bacterial Microbiomes in Oropharynx during Infection and Recovery of COVID-19 Omicron Variant
Source: PLoS Pathog. 2024 Apr 3;20(4):e1012075. doi: 10.1371/journal.ppat.1012075 (PMC10990182; doi:10.1371/journal.ppat.1012075)

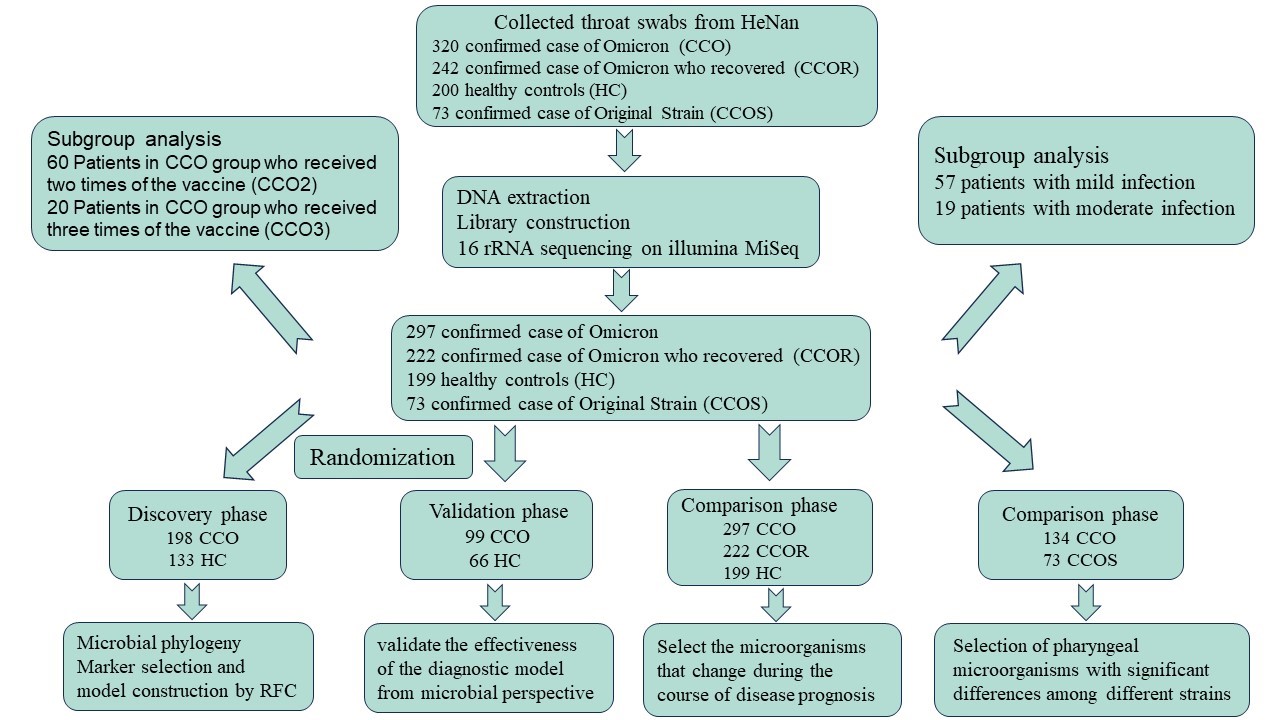

Supplement: S1 Fig — A total of 835 oropharyngeal specimens were collected from Henan Province, China. After screening, 791 samples were sequenced by 16S rRNA MiSeq, including 297 CCO, 222 CCOR, 199 HC and 73 CCOS. CCO, confirmed cases of COVID-19 Omicron variant; CCO2, patients in CCO group who received two times of the vaccine; CCO3, patients in CCO group who received three times of the vaccine; CCOR, confirmed cases of COVID-19 Omicron who recovered; HC, healthy controls; CCOS, confirmed cases of COVID-19 Original strain. RFC, random forest classifier. (JPG) [file ppat.1012075.s001.jpg]

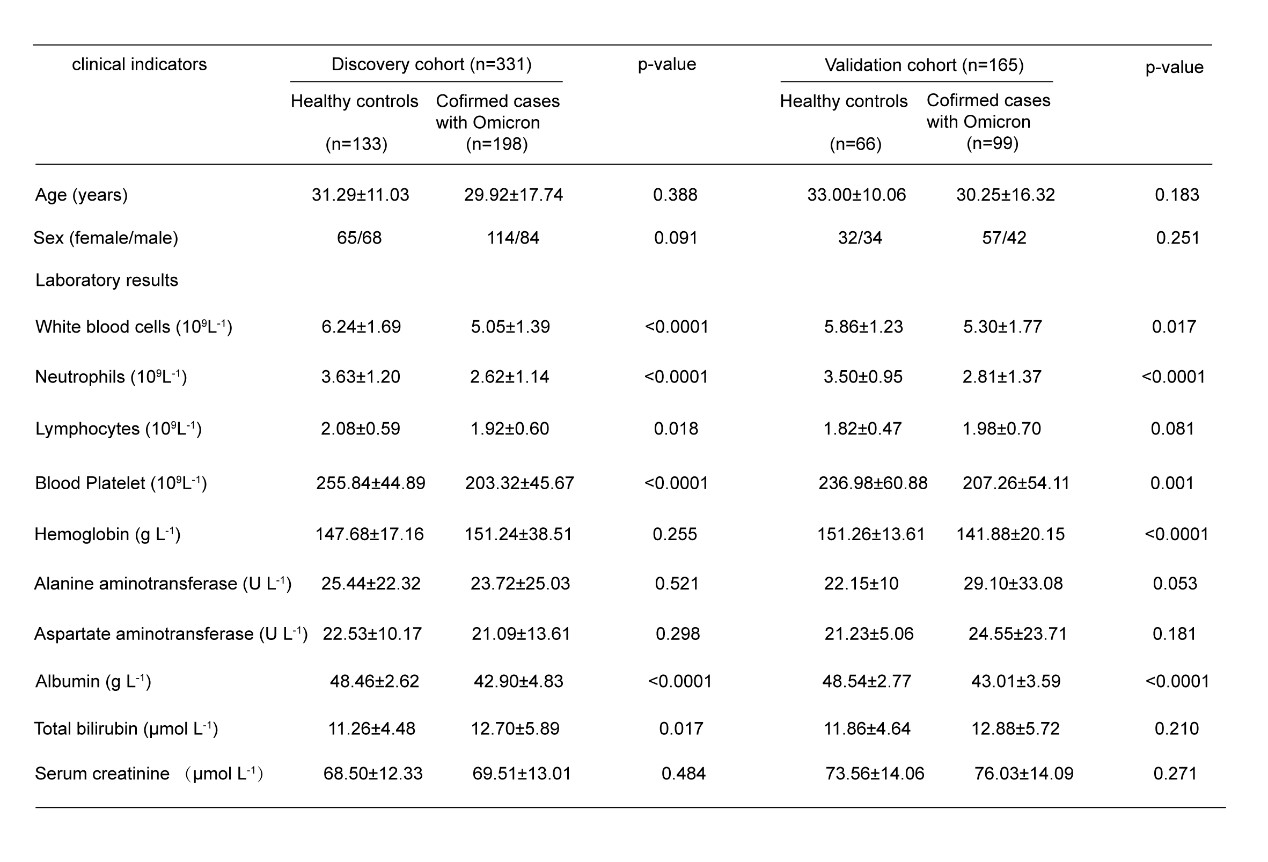

Supplement: S2 Fig — We presented continuous variables as the means (standard deviations) and categorical variables as percentages. Differences between subjects with SARS-CoV-2 Omicron strain infection (n = 198, n = 99) and healthy controls (n = 133, n = 66) were carried out by using Student’s t-test for normally distributed continuous variables, the Wilcoxon rank-sum test for non-normally distributed continuous variables, and the chi-square test for categorical variables. Statistical significance was defined by p < 0.05 (two-tailed). (JPG) [file ppat.1012075.s002.jpg]

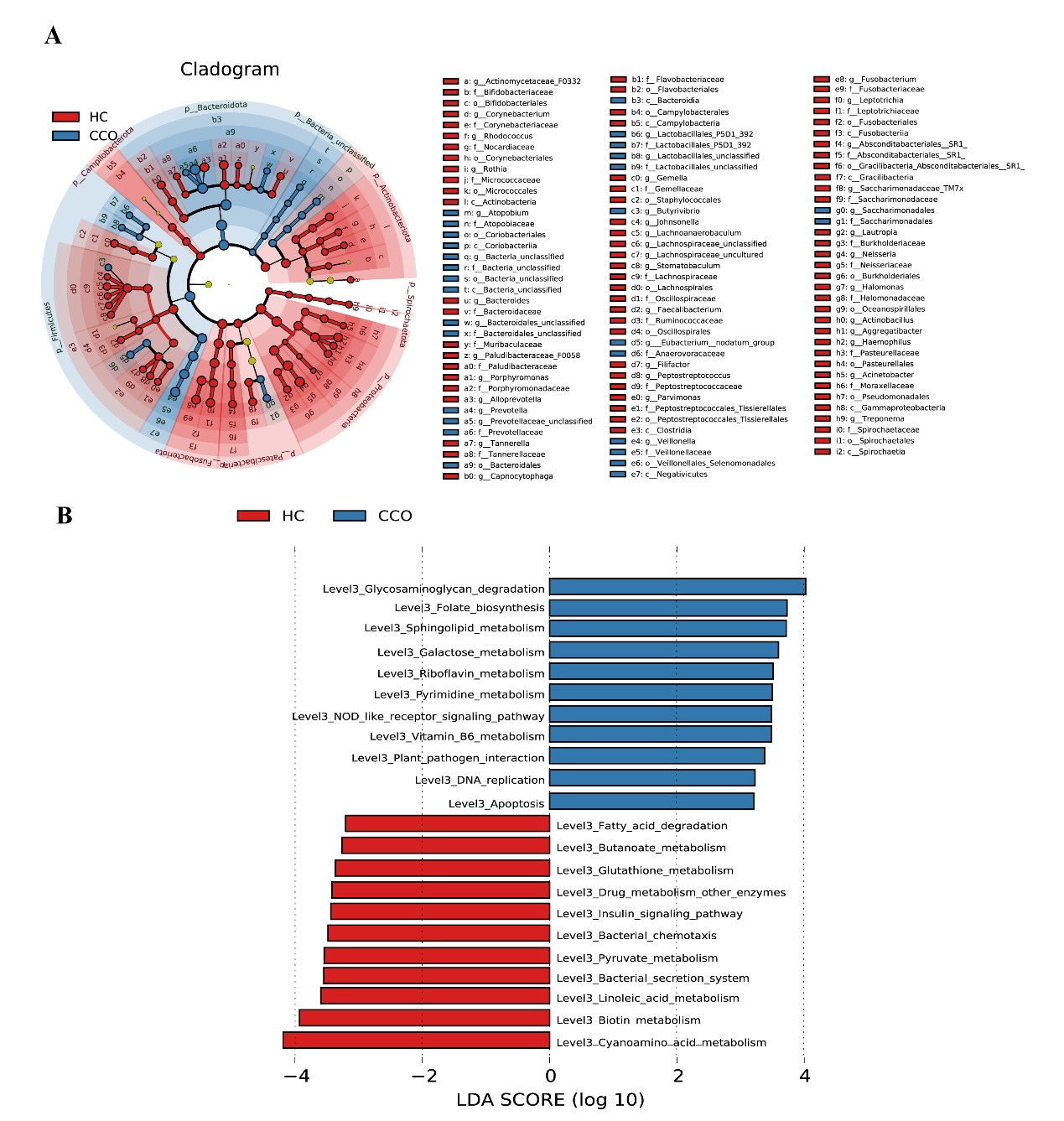

Supplement: S3 Fig — A) The cladogram, representing oropharyngeal microbial structure and their predominant bacteria, revealed the greatest differences in different taxa between CCO group (n = 198) and HC group (n = 133). B) Based on the LDA selection, 47 gene functions were significantly enhanced in CCO group and 42 gene functions in HC group. (p<0.05, LDA>3). CCO, confirmed cases of Omicron variant; HC, healthy controls; LEfSe, linear discriminant analysis (LDA) effect size (Only part of the bacteria is displayed. Please see the S12 Table for details). (JPG) [file ppat.1012075.s003.jpg]

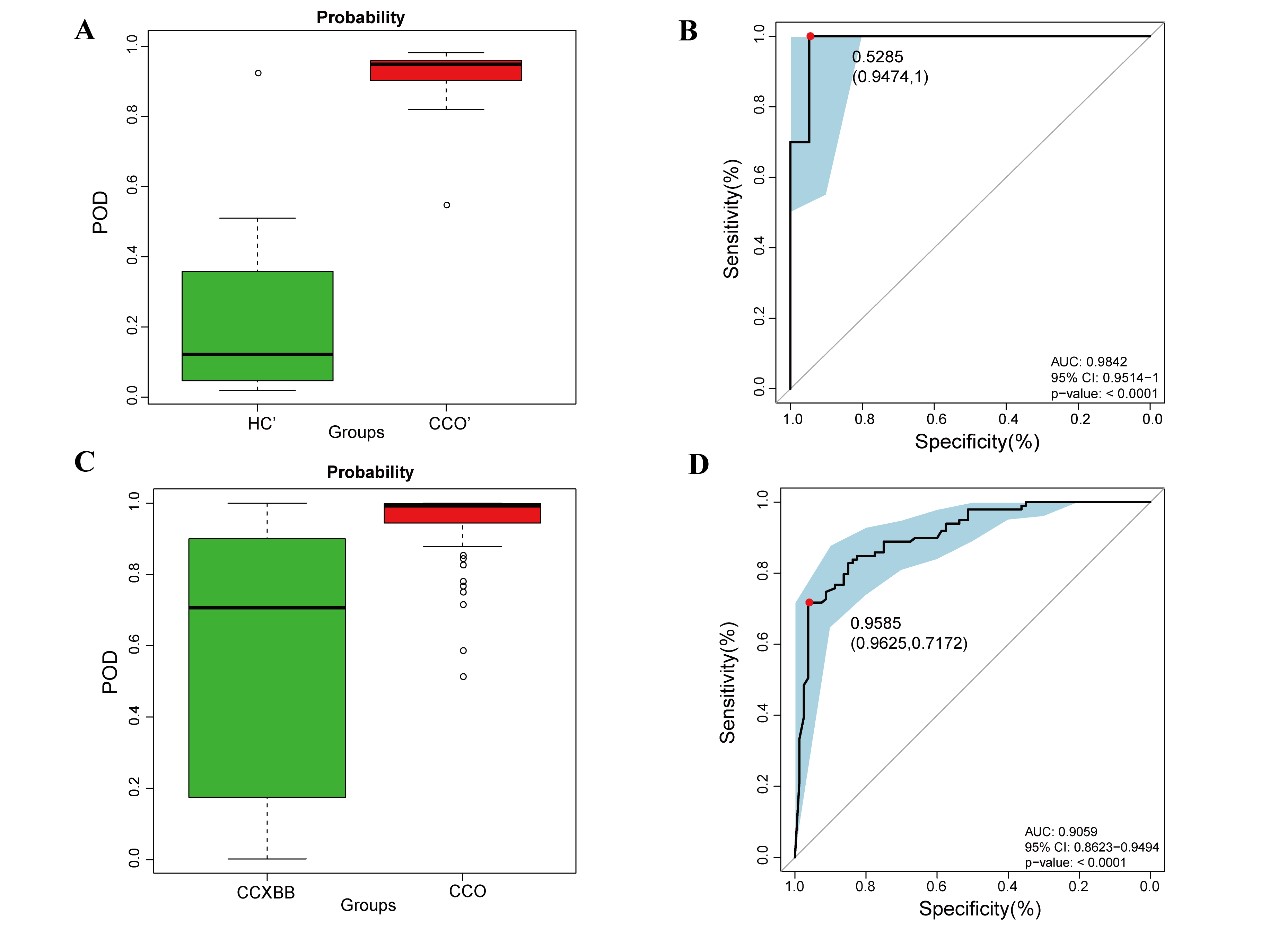

Supplement: S4 Fig — A) The POD value was significantly higher in CCO’ group (n = 20) compared with that in HC’ group (n = 20) in the verification cohort. B) The POD value achieved an AUC of 98.42% (95% CI 95.14% to100%, p<0.0001) between CCO’ group (n = 20) versus HC’ group (n = 20) in the verification cohort. C) The POD value was significantly higher in CCO group (n = 99) compared with that in CCXBB group (n = 80) in the verification cohort. D) The POD value achieved an AUC of 90.59% (95% CI 86.23% to94.94%, p<0.0001) between CCO group (n = 99) versus CCXBB group (n = 80) in the verification cohort. CCO, confirmed cases of COVID-19 Omicron variant; HC, healthy controls; CCO’, Cross-age verification cohort of COVID-19 Omicron variant; HC’, Cross-age verification cohort of healthy controls; CCXXB, confirmed cases of Omicron subvariants XBB.1.5; OTUs, operational taxonomy units; POD, probability of disease; AUC, area under the curve; centerline, median; box limits, upper and lower quartiles; error bars, 95% CI. (JPG) [file ppat.1012075.s004.jpg]

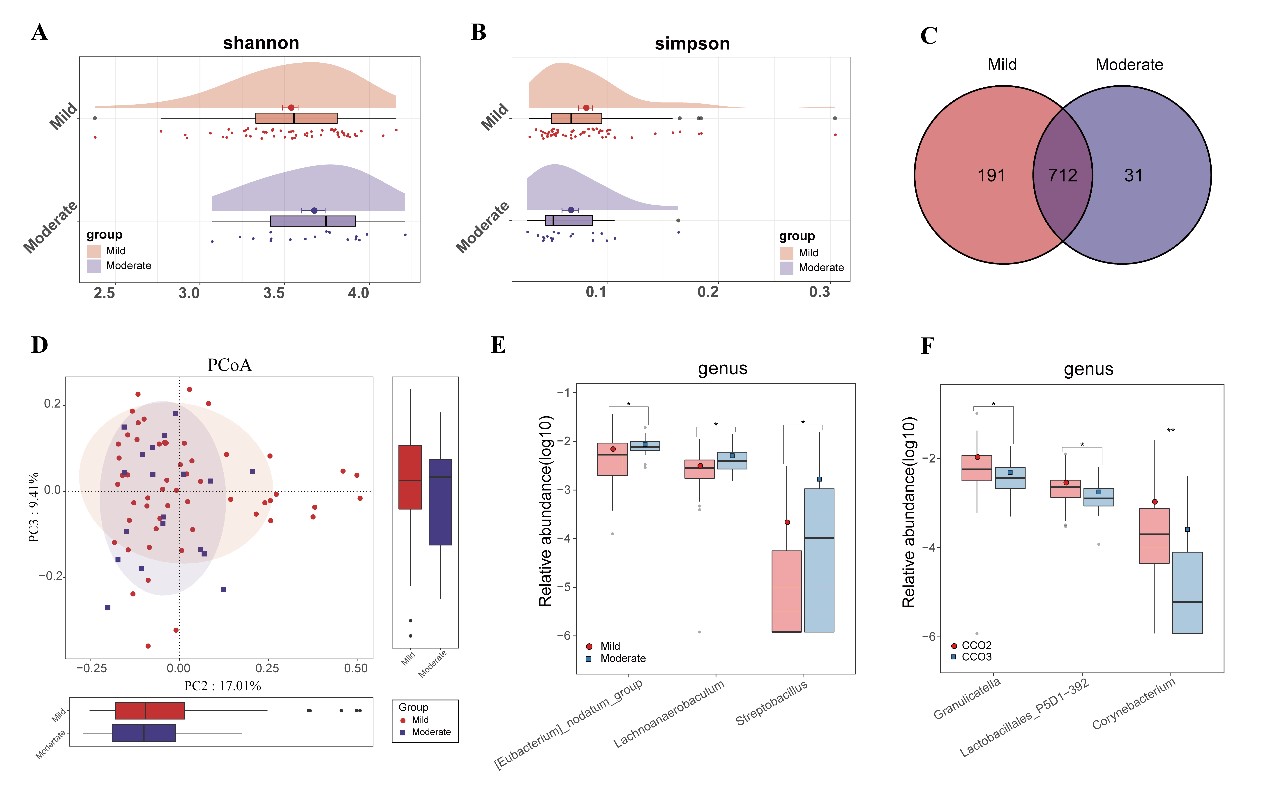

Supplement: S5 Fig — A) Shannon index showed that there was no significant difference in microbial α-diversity between moderate and mild patients. (p>0.05). B) Simpson index showed that there was no significant difference in microbial α-diversity between moderate and mild patients. (p>0.05). C) A Venn diagram displaying the overlaps between groups showed that 712 of 934 OTUs were shared in Mild and Moderate groups, while 31 of 934 OTUs were unique to the Moderate group. D) The PCoA based on OTU distribution showed the oropharyngeal taxonomic composition was significantly different between the two groups. E) Compared with Mild group, 3 genera increased significantly in Moderate group. F) Compared with CCO2 group, 3 genera decreased significantly in CCO3 group. *p<0.05, **p<0.01, ***p<0.001. CCO, confirmed cases of COVID-19 Omicron variant; HC, healthy controls; CCO2, patients in CCO group who received two times of the vaccine; CCO3, patients in CCO group who received three times of the vaccine; OTUs, operational taxonomy units; PCoA, principal coordinate analysis; centerline, median; box limits, upper and lower quartiles; error bars, 95% CI. (JPG) [file ppat.1012075.s005.jpg]

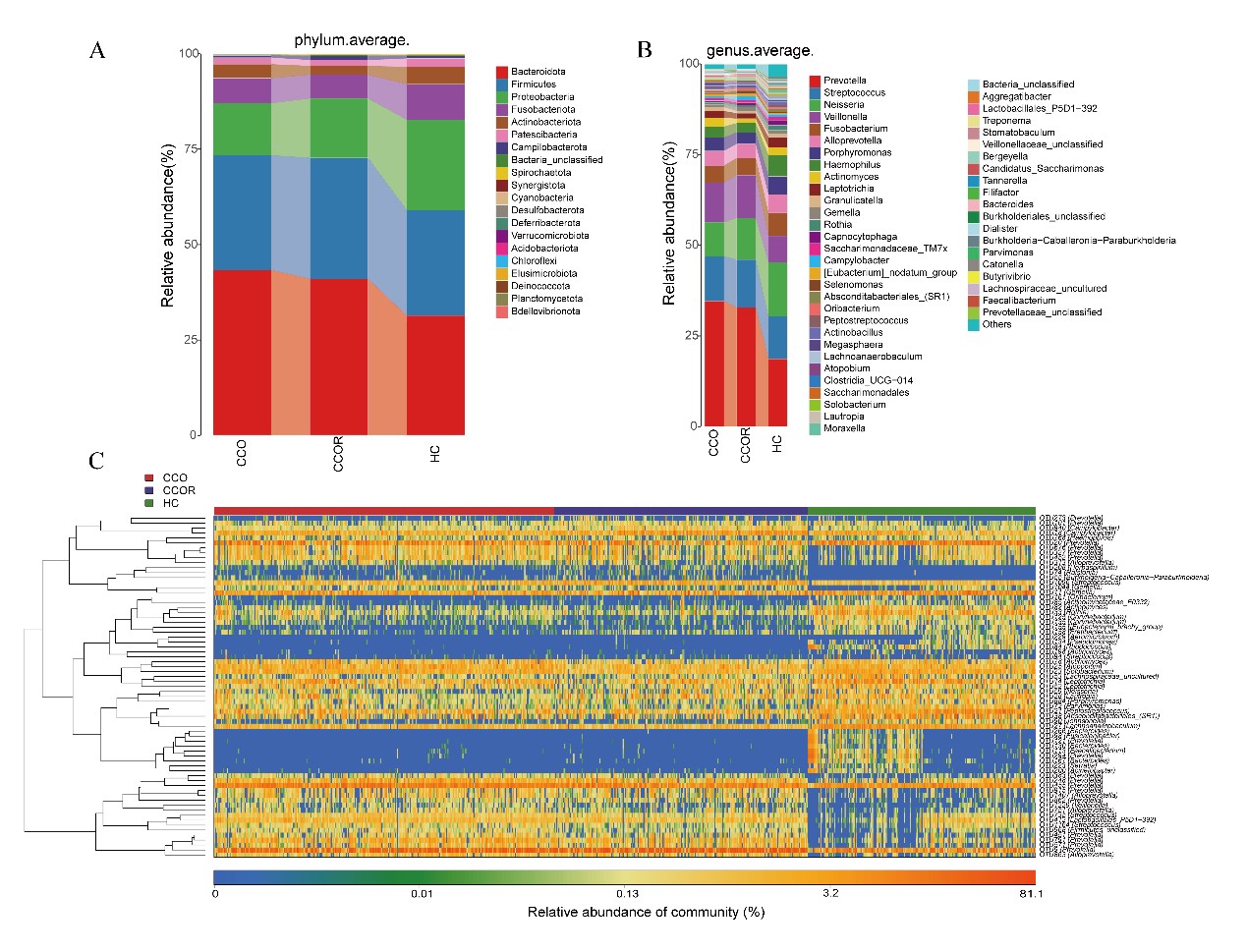

Supplement: S6 Fig — A) Average compositions and relative abundance of the bacterial community at the phylum level among CCO, CCOR and HC groups. B) Average compositions and relative abundance of the bacterial community at the genus level among CCO, CCOR and HC groups. C) Heatmap showed the relative abundances of differential OTUs for each sample among CCO, CCOR and HC groups. CCO, confirmed cases of COVID-19 Omicron variant; CCOR, confirmed cases of omicron who recovered; HC, healthy controls; OTUs, operational taxonomy units; centerline, median; box limits, upper and lower quartiles; circle or square symbol, mean; error bars, 95% CI. (JPG) [file ppat.1012075.s006.jpg]

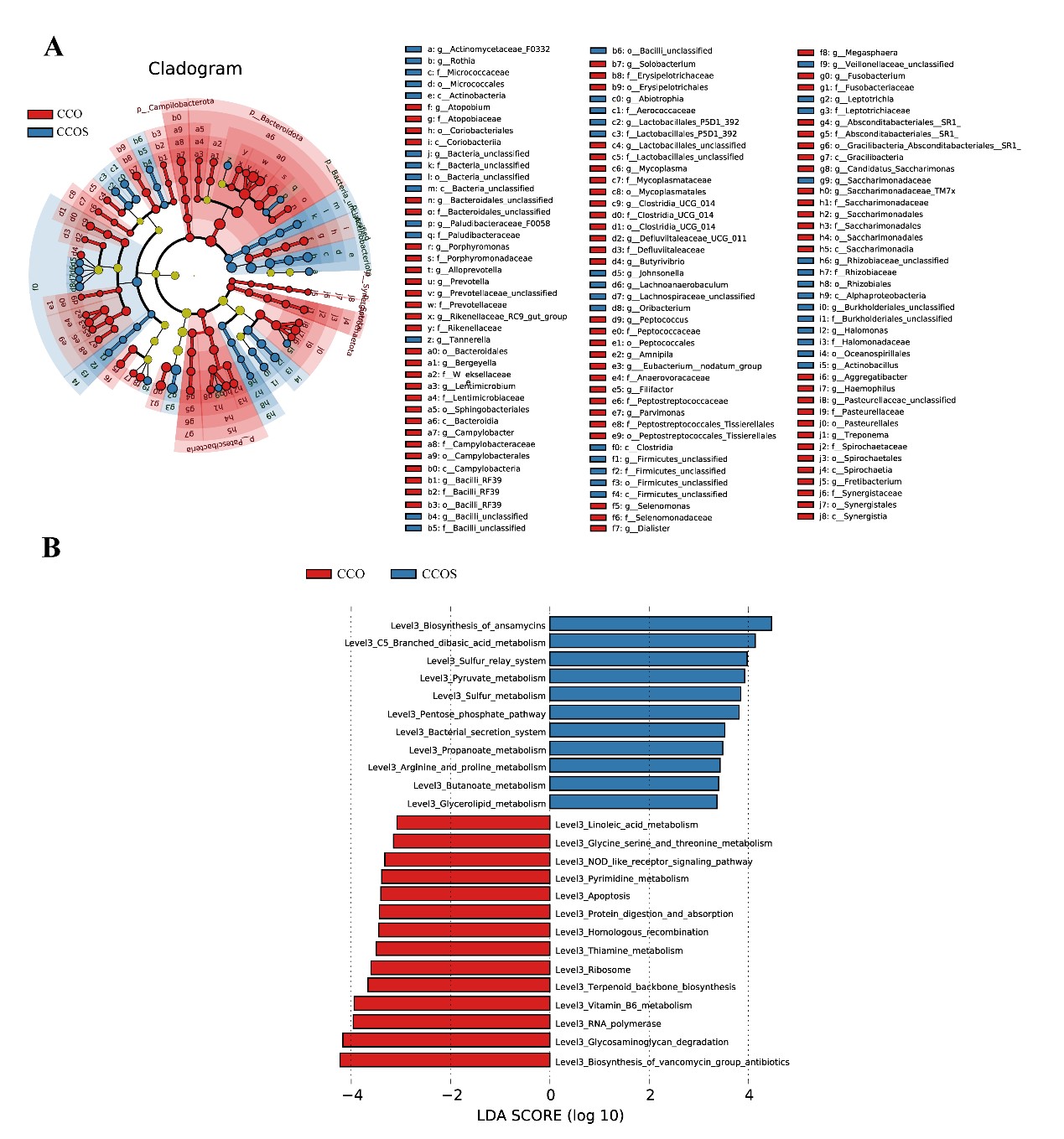

Supplement: S7 Fig — A) The cladogram, representing oropharyngeal microbial structure and their predominant bacteria, revealed the greatest differences in different taxa between CCO group (n = 134) and CCOS group (n = 73). B) Based on the LDA selection, 41 gene functions were significantly enhanced in CCO group and 33 gene functions in CCOS group. (p<0.05, LDA>3). CCO, confirmed cases of Omicron variant; CCOS, confirmed cases of original strain; LEfSe, linear discriminant analysis (LDA) effect size (Only part of the bacteria is displayed. Please see the S37 Table for details). (JPG) [file ppat.1012075.s007.jpg]
