# Supplementary material for: Dynamic changes of Bacterial Microbiomes in Oropharynx during Infection and Recovery of COVID-19 Omicron Variant
Source: PLoS Pathog. 2024 Apr 3;20(4):e1012075. doi: 10.1371/journal.ppat.1012075 (PMC10990182; doi:10.1371/journal.ppat.1012075)
Supplement: S1 Supplementary Methods — (DOCX) [file ppat.1012075.s046.docx]

**Dynamic changes of Bacterial Microbiomes in Oropharynx during Infection and Recovery of COVID-19 Omicron Variant**

**Supplementary method**

**Diagnostic, inclusion, and exclusion criteria**

Diagnostic criteria are based on the “COVID-19 diagnosis and treatment program trial V.9 guidelines” issued by the National Health Commission of the People’s Republic of China.

1. Suspected case

There is any one of the following epidemiological history and conforms to any two of the clinical manifestations.

If there is no clear history of epidemiology, it conforms to 3 of the clinical manifestations, or conforms to any 2 of the clinical manifestations, and novel coronavirus's specific IgM antibody is positive (those who have recently been vaccinated with novel coronavirus vaccine are not taken as a reference index).

(1) History of Epidemiology

①Travel history or residence history in the community where the case was reported within 14 days prior to onset of illness.

②A history of contact with novel coronavirus infection within 14 days before the onset of the disease.

③Had contact with patients with fever or respiratory symptoms from the reported community within 14 days before the onset of the disease.

④Cluster onset (2 or more cases of fever and / or respiratory symptoms within 14 days in a small area such as home, office, school, class, etc.).

(2) Clinical manifestation

COVID-19-related clinical manifestations such as fever and / or respiratory symptoms.

②With the above-mentioned imaging features of COVID-19.

③In the early stage of the disease, the total leukocyte count was normal or decreased, and the lymphocyte count was normal or decreased.

2. Confirmed case

The suspected case has one of the following etiological or serological evidence:

(1) Novel coronavirus tested positive for nucleic acid.

(2) Novel ①coronavirus specific IgM antibody and IgG antibody were positive in those who were not vaccinated with novel coronavirus vaccine.

3.Rehabilitation case

①The body temperature returned to normal for more than 3 days.

②Respiratory symptoms obviously improved.

③Pulmonary imaging showed that acute exudative lesions were significantly improved.

④The Ct values of N gene and ORF gene detected by novel coronavirus nucleic acid were ≥ 35 for two consecutive times (RT-PCR method, the limit value was 40, sampling time was at least 24 hours), or novel coronavirus nucleic acid test was negative for two consecutive times (RT-PCR method, the cutoff value was less than 35, sampling time was at least 24 hours).

Those who meet the above conditions can be discharged.

All registered persons are examined by a professional stomatologist to ensure the health of the mouth and gums. The samples of all healthy volunteers are from the physical examination Department of the first affiliated Hospital of Zhengzhou University. The inclusion criteria can be referred to our previous study. The exclusion criteria included diabetes, obesity, hypertension, metabolic syndrome, irritable bowel syndrome, non-alcoholic fatty liver disease, liver cirrhosis and celiac disease. Individuals who received antibiotics and / or probiotic treatment within 8 weeks prior to registration were also excluded.

**Enrollment process**

COVID-19 designated hospital in Henan Province has set up a number of suspected case wards and confirmed case wards. The suspected case ward is used to treat patients who meet the conditions of suspected cases in the guidelines for diagnosis and treatment. After admission, professionals collect throat swab samples from related patients for RT-PCR nucleic acid testing. If the nucleic acid is positive, it is immediately transferred to the confirmed case ward. Our investigators will then assess whether the patient meets our registration criteria. If the conditions are met, the patient will be included in the confirmed case group after signing the informed consent. In the end, our investigators screened and recruited eligible patients. The patients who met the conditions of rehabilitation cases in the diagnosis and treatment guidelines were transferred to the out-of-hospital isolation area and observed for 15 days. If the nucleic acid test is not negative during this period, the recovered patient can leave the isolation area. Our investigators will collect samples from them two days before discharge.

**Instructions for excluding confounding factors**

Our study controlled for confounding following factors:

(1) Regarding antibiotic usage and OTC drugs, the collection of these samples was in the stage of epidemic control in China, and the government has been carrying out epidemiological investigations of the COVID-19, and once cold-like symptoms appear, they are directly sent to designated hospitals for nucleic acid testing, and over-the-counter drugs such as antipyretics are under control during the epidemic and cannot be purchased by individuals. After the patient is confirmed to be positive for nucleic acid, he will be isolated to the hospital within one day. A throat swab sample is taken immediately after admission. The treatment plan of the patients after admission was based on the "Diagnosis and Treatment Plan for COVID-19 (Trial Ninth Edition)", and antiviral therapy combined with symptomatic supportive care was adopted, and antibiotics and hormones were not used.

(2) Regarding geographical variations, all samples were collected within Henan Province.

(3) Considering dietary differences, as patients were treated under centralized isolation, dietary conditions were largely uniform. Moreover, the eating habits of people in the same area are basically similar.

**PCR amplification**

We continue the previous operation and continue to use primers targeting the hypervariable V3-V4 region of the 16S rRNA gene to amplify the extracted DNA samples. The forward primer was 5’-ACTCCTACGGGAGGCAGCA-3’ and the reverse primer was 5’-GGACTACHVGGGTWTCTAA T-3’. The PCR reaction system including 0.4μL of Fast Pfu polymerase, 2μL of 2.5 mM dNTP (TransGen Biotech, Beijing, China), 4μL of 5×Fast Pfu buffer, 0.8μL of each primer (5μM), and 10 ng of template DNA. Each sample were carried out four reactions using a PCR machine (ABI GeneAmp 9700): 95 ° C for 2 minutes, 95 ° C for 30 s, 55 ° C for 30 s, 72 ° C for 30 s, repeating 30 cycles and finally at 72 ° C for 5 minutes. Agarose gel (Axygen Biosciences, Union City, CA) was used to separate, extract and purify the PCR products, and the products were quantified using a fluorescence assay kit (Quant-iT PicoGreen, Invitrogen).

**Sequence data process**

Process the amplified readings through the following steps: (i) overlap pair end sequenced reads of each library using FLASH v1.2.10 software. (ii) use the customization of each program to perform more specific quality control on overlapping reads generated by FLASH 1) Ambiguous bases (N); 2) the mismatch rate in the overlap region was no more than 0.05; 3) mismatches in barcode and primer regions were not approved; (iii) de-multiplexed and assigned reads into different samples according to barcodes; (iv) detect and remove the chimeric sequences by UCHIME version 4.2.40 (version microbiome util-r20110519, http://drive5.com/uchime/gold.fa) to match Operational Taxonomy Units (OTUs).

**Construction of probability of disease (POD)**

We used the abundance profile of the optimal OTUs markers in the discovery cohort to conduct fivefold cross-validation on a random forest model (R 3.4.1, random Forest 4.6-12 package). Then we acquired the cross-validation error curve through five trials of the fivefold cross-validation. The point with the minimum cross-validation error was defined as the cut-off point through the minimum error plus the standard deviation (SD). The sets of OTU markers with the error less than the cut-off value were listed and the set with the smallest number of OTUs were defined as the optimal set. The POD exponents in both the discovery queue and the verification queue are calculated using the optimal OTUs set. The model is evaluated by receiver operating characteristic (ROC) curve (R3.3.0 proc software package), and AUC is used to show ROC effect.
