# Supplementary material for: Dynamic changes of Bacterial Microbiomes in Oropharynx during Infection and Recovery of COVID-19 Omicron Variant
Source: PLoS Pathog. 2024 Apr 3;20(4):e1012075. doi: 10.1371/journal.ppat.1012075 (PMC10990182; doi:10.1371/journal.ppat.1012075)
Supplement: Attachment — Submitted filename: renamed_6a0b7.docx [file ppat.1012075.s047.docx]

December 28, 2023

Ashley L. St. John

Academic Editor

PLOS Pathogens

Ana Fernandez-Sesma

Section Editor

PLOS Pathogens

Kasturi Haldar

Editor-in-Chief

PLOS Pathogens

RE: Dynamic changes of Bacterial Microbiomes in Oropharyngeal during Infection and Recovery of COVID-19 Omicron Variety (PPATHOGENS-D-23-01358)

Dear Editor:

Thank you very much for your help and great contribution on our manuscript entitled "Dynamic changes of Bacterial Microbiomes in Oropharyngeal during Infection and Recovery of COVID-19 Omicron Variety" (PPATHOGENS-D-23-01358).

We have made a point-to-point response according to the comments from editors and reviewers. The details can be found as follows. We have revised the manuscript, and carefully proofread the whole manuscript. The revised manuscript has been edited and polished for proper English language, grammar, punctuation, spelling, and overall style.

We resubmit the revised manuscript, including figures and the point-to-point response letter.

**COMMENTS TO Editor**

*The authors should address the reviewers' concerns regarding* ***the comparisons to previous studies*** *and to* ***other variants****, potentially by* ***adding further analysis****. Also, consider* ***changing the word "variety" to "variant"*** *in the title.* ***The readability of certain tables, e.g. S2*** *(which might be better suited as a supplementary data set rather than a table) needs to be improved. There are* ***whole pages of table without any contents****, so the quality of the supplemental section in general needs to be improved to publication quality. Claims of* ***"first time" should be removed*** *from the discussion. Much of the speculation on mechanisms in the discussion is unsupported and should be* ***toned down*** *in the absence of any supporting data. Further* ***evidence towards the influence of the short chain fatty acids*** *derived from these bacteria on* ***SARS-CoV-2 infection severity*** *would improve the manuscript.*

**Author response**: Thank you very much for constructive comments.

1. Comparison with the article “Metagenomic analysis reveals oropharyngeal microbiota alterations in patients with COVID-19” of Ma et al.

Similarity: (1) The results revealed a distinct oropharyngeal microbiota composition in the COVID-19 patients. (2) Based on the relative abundance of the oropharyngeal microbiome, the study built a microbial classifier to distinguish COVID-19 patients from healthy controls. (3) Genetic analysis of metabolic pathways related to oropharyngeal microbiomes in patients with COVID-19 infection. (4) A subgroup analysis was carried out on the severity of the disease.

Differences: (1) The types of strains studied are different. Ma et al. studied the COVID-19 original strains, and our study is COVID-19 Omicron variant. (2) The characteristics of oropharyngeal microbiome of the infection recovery of COVID-19 Omicron variant were described in our study. (3) Our study also included the analysis of the characteristics of oropharyngeal microbiome of patients with different times of vaccination. (4) we compared and analyzed the oropharyngeal microbiomes of Omicron variant and the original strain.

2. Comparison with the article “Oral Microbiome Dysbiosis Is Associated With Symptoms Severity and Local Immune/Inflammatory Response in COVID-19 Patients: A Cross-Sectional Study” of Irene Soffritti et al.

Similarity: (1) The results revealed a distinct microbiota composition in the COVID-19 patients. (2) A subgroup analysis was carried out on the severity of the disease.

Differences: (1) The types of strains studied are different. Irene Soffritti et al. studied the COVID-19 original strains, and our study is COVID-19 Omicron variant. (2) Different clinical samples are used. Irene Soffritti et al. used oral rinse samples, we used pharyngeal swab sample. (3) The characteristics of oropharyngeal microbiome of the infection recovery of COVID-19 Omicron variant were described in our study. (4) Based on the relative abundance of the oropharyngeal microbiome, the study built a microbial classifier to distinguish COVID-19 patients from healthy controls. (5) Our study also included the analysis of the characteristics of oropharyngeal microbiome of patients with different times of vaccination. (6) we compared and analyzed the oropharyngeal microbiomes of Omicron variant and the original strain.

3. Comparison with the article “Dysbiosis of Oral and Gut Microbiomes in SARS-CoV-2 Infected Patients in Bangladesh: Elucidating the Role of Opportunistic Gut Microbes” of S. M. Rafiqul Islam et al.

Similarity: (1) The results revealed a distinct microbiota composition in the COVID-19 patients. (2) Genetic analysis of metabolic pathways related to oropharyngeal microbiomes in patients with COVID-19 infection.

Differences: (1) The types of strains studied are different. S. M. Rafiqul Islam et al. studied the COVID-19 original strains, and our study is COVID-19 Omicron variant. (2) Different clinical samples are used. S. M. Rafiqul Islam et al. used stool and saliva samples, we used pharyngeal swab sample. (3) Based on the relative abundance of the oropharyngeal microbiome, the study built a microbial classifier to distinguish COVID-19 patients from healthy controls. (4) The characteristics of oropharyngeal microbiome of the infection recovery of COVID-19 Omicron variant were described in our study. (5) Our study also included the analysis of the characteristics of oropharyngeal microbiome of patients with different times of vaccination. (6) we compared and analyzed the oropharyngeal microbiomes of Omicron variant and the original strain.

Based on your suggestions, we have incorporated additional analyses: "Oropharyngeal microbiome analysis of different SARS-CoV-2 infection severity " and "Oropharyngeal microbiome analysis from individuals vaccinated with two doses versus three doses." Furthermore, we introduced two new validation cohorts: the middle-aged and elderly cohort and the XBB.1.5 variant. Our findings demonstrate the applicability of our diagnostic model in the middle-aged and elderly populations, successfully distinguishing between two distinct variants of Omicron.

We have changed the word "variety" to "variant" in the title. Moreover, we have enhanced all supplementary datasets, improving the overall quality to prevent entirely blank pages in the tables. Additionally, we changed the tables regarding clinical data of the infected individuals from the previous version to the picture and relocated it to the supplementary pictures. In addition, we have removed the claims of "first time" from the discussion in this study and toned down speculative aspects in the discussion. We have added moderate and unbiased adjectives to the discussion.

We utilized propensity score matching to control for clinically relevant confounders and then included 57 mild and 19 moderate Omicron patients. We compared and analyzed the differences in the oropharyngeal microbiota between these two groups. Additionally, we conducted KEGG metabolic pathway analysis aiming to identify evidence at the genetic level that short-chain fatty acids associated with microbiota affect disease severity. However, the microbial and genetic functional differences between the groups weren't prominent, possibly due to inadequate sample size or the comparatively lower virulence of the BA.5.2 Omicron variant. Consequently, we did not ascertain evidence linking short-chain fatty acids to the severity of COVID-19 infection. Nevertheless, we referenced findings from other studies regarding the association of short-chain fatty acids with the severity of coronavirus infections (non-Omicron strains), enhancing the breadth of our paper.

**COMMENTS TO AUTHOR:**

**Reviewer #1**:

**Part I – Summary**

*In this study the authors characterize oropharyngeal microbiomes of individuals infected by the Omicron variant to show that their microbiome vary from that of the original strain. The authors state that this is important because the oropharyngeal microbiome plays an important role in the severity of the disease. They then go on to construct a panel of a non-invasive microbial markers which could potentially be used for diagnostic purposes.*

**Author response:** We sincerely thank you for your valuable feedback that we have used to improve the quality of our manuscript. The reviewer comments are laid out below in blue text and specific concerns have been numbered. Our response is given in black text.

*The sample size of this study is clearly a strength however* ***the novelty and significance are lowered*** *given that another study by Zhang et al., also show (albeit with a smaller sample size) that the oropharyngeal microbiome diversity is reduced and less diverse in SARS-CoV2 patients. The novelty of the study, as stated by the authors, is that the Omicron variant had different microbiomes from the original strain. They also include a cohort of patients that have recovered from the Omicron variant.*

*In my opinion the study is executed well (the authors also previously publish this study design for the original strain of SARS-CoV2, Gao et al., 2021). However, I think the strength of the study could be improved by* ***adding further mechanistic study*** *to account for the differences in oropharyngeal microbiomes of individuals infected with Omicron.*

**Author response:** We thank you very much for pointing out this issue. Compared to Zhang et al article, the large sample size is our advantage. In order to increase the innovation and opinions of the article, we have made major revisions to the article according with your suggestion. The specific revisions and innovations are as follows:

(1) Our study delineated the distinctive oropharyngeal microbiota features associated with the Omicron, identifying bacterial variations compared to healthy individuals and reporting bacteria that gradually change with disease recovery. These alterations might be relevant to the disease course and post-COVID sequelae. Utilizing propensity score matching (PSM) to eliminate confounding factors, we specifically analyzed 57 mild and 19 moderate infection cases, identifying key bacteria influencing disease severity. This discovery provides new targets for targeted microbiome interventions to alleviate disease progression.

(2) We established a non-invasive diagnostic model through training and validation sets. To demonstrate the universality of this non-invasive diagnostic approach, we incorporated a cross-age validation cohort. This confirmed that the model maintains a high diagnostic efficiency across different age groups. Furthermore, to validate our diagnostic markers as authentic indicators of Omicron subvariants BA.5.2, we included another Omicron subvariants XBB.1.5, finding that the diagnostic model accurately distinguished between Omicron subvariants BA.5.2 and XBB.1.5. This demonstrates the microbial markers' specificity for Omicron subvariants and their clinical applicability, significantly enhancing the strengths of this project.

(3) This project analysed of the microbial differences in the microbiota between the original and Omicron variant. It identified changes in the post-infection microbiota associated with Omicron variant compared to the original strain, highlighting variations in bacterial abundance. This reveals patterns of microbial changes due to viral mutations and their dynamics following variant strain infections.

**Part II – Major Issues: Key Experiments Required for Acceptance**

*1. Have the authors read Ma, S., Zhang, F., Zhou, F. et al. Metagenomic analysis reveals oropharyngeal microbiota alterations in patients with COVID-19. Sig Transduct Target Ther 6, 191 (2021). https://doi.org/10.1038/s41392-021-00614-3. This is not sited in the current version of the manuscript and should be given the similarities of this study with the authors current manuscript. The authors should also add in a discussion comparing the findings of both papers.*

**Author response:** Thank you very much for constructive comments.

(1) The study of Ma et al described the characteristics of oropharyngeal microbiome of COVID-19 original strain, and our study described the characteristics of oropharyngeal microbiome of COVID-19 Omicron variant. We found that the patients infected with Omicron variant had microbiomes imbalance in oropharynx, and the microbial diversity was significantly less than that in healthy individuals. Compared with HC group, some conditional pathogens such as *Prevotella* and *Veillonella* were significantly increased in CCO group, while some acidogenic bacteria such as *Fusobacterium* and *Alloprevotella*, which regulate metabolic function, were significantly decreased in CCO group. More importantly, we also compared the oropharyngeal microbial characteristics of the Omicron variant and the original strain. This provides clues for early diagnosis and disease treatment among different strains of novel coronavirus.

(2) The characteristics of oropharyngeal microbiome of the infection recovery of COVID-19 were not described in the study of Ma et al. We found that with the recovery of the infection of Omicron variant, 4 genera such as *Actinobacillus* increased gradually, while 7 genera such as *Atopobium* and *Lactobacillales* decreased gradually. The above results suggest that these bacteria may be involved in the progress or recovery of infection.

(3) Ma et al. identified a microbial genus or species classifier distinguishing COVID-19 patients from flu patients and healthy controls. we also constructed a diagnostic model based on 6 optimal oropharyngeal OTUs in the discovery cohort, and achieved extremely high diagnostic performance in the verification cohort. Not only that, our diagnostic model also maintains high diagnostic efficiency in cross-age verification cohorts, and can be accurately distinguished from another Omicron subtype XBB.1.5.

(4) Our study also included the analysis of the characteristics of oropharyngeal microbiome of patients with different times of vaccination. The results suggest that the oropharyngeal microbiota may be related to the efficacy of COVID-19 vaccine, and oropharyngeal dysbiosis may reduce the COVID-19 vaccine efficacy and persistence.

*2. Can the authors sample patients with another variant of less severity to strengthen the notion that these are bonafide markers of the Omicron variant?*

**Author response:** Thank you very much for constructive comments. Based on your advice, we made revisions to the manuscript. We collected oropharyngeal swab samples from individuals infected with the Omicron subvariant, XBB.1.5 strain. Using PSM to mitigate confounding factors like gender and age, we included a final cohort of 80 individuals infected with the Omicron XBB.1.5 strain. Subsequently, we applied the previously constructed diagnostic model to this new cohort. The results indicated that the diagnostic model based on oropharyngeal microbiota accurately distinguished between the Omicron BA.5.2 and XBB.1.5 strains, and the AUC value was 90.59% (95% CI 86.23% to 94.94%, p<0.0001)), further demonstrating the specificity of this diagnostic model.

*3.I find this manuscript is* ***very similar to their previous work*** *on the original strain Gao et al., 2021 and I feel that this manuscript would be strengthened by further research into the* ***molecular mechanism and/or possible pathways and targets of the microbiome*** *which can be related to the Omicron clinical severity or outcome.*

**Author response:** Thank you very much for your issue. Compared to our previous work, we have made major revisions to the article according with your suggestion, in order to increase the innovation and opinions of the article. (1) Compared to previous articles, our model can be applied to people of different ages. In addition, our model is able to accurately distinguish between the Omicron subvariants BA.5.2 and XBB.1.5, suggesting that the model is more universal and specific. (2) In this project, a number of subgroup analyses, such as the original variant and the Omicron variant, mild and common infection, and 2-times and 3-times vaccines, were performed to clarify the differences in oropharyngeal microbiota between the original variant and the Omicron variant, to identify differential microbiota that may affect disease severity, and to exlpain the impact of the number of vaccinations on the microbiota.

According to your recommendations, we utilized propensity score matching to eliminate confounding factors such as age, gender, white blood cells, neutrophils, lymphocytes, hemoglobin, alanine aminotransferase, aspartate aminotransferase, albumin, total bilirubin, urea, and number of vaccine doses administered. Finally, we included 57 cases of mild infection and 19 cases of moderate infection. We identified key bacteria, including *Streptobacillus*, *Lachnoanaerobaculum*, and *Eubacterium nodatum*, associated with the severity of the disease. Subsequently, we conducted KEGG gene functional analysis to explore potential metabolic pathways or molecules influencing disease severity. Results indicated a significant enrichment of the Threonine biosynthesis pathway in mild patients, while pathways like Allose transport system and Trehalose maltose transport system were notably enriched in moderate patients (details in the following figure). These findings offer novel targets for mitigating disease progression through targeted microbiota interventions.

**Part III – Minor Issues: Editorial and Data Presentation Modifications**

*1. bacterial strains should be* ***italicized****.*

**Author response:** Thank you very much for constructive comments. All bacterial strains mentioned in the manuscript have been changed to italics. The details could be found in the revised manuscript.

*2. Figure 3 legends should include* ***a separate reference to each graph*** *within figure 3*

**Author response:** Thank you very much for constructive comments. We have included a separate reference to each graph within figure 3. The details could be found in the revised manuscript.

**Reviewer #2**:

**Part I – Summary**

*Scope and Relevance: The investigation into the oropharyngeal microbiome's role in the context of COVID-19 infections, particularly with the Omicron variant, is both timely and relevant. As the pandemic evolves and new strains emerge, understanding these nuances becomes crucial for diagnostics and therapeutic interventions.*

**Author response:** We sincerely thank you for your valuable feedback.

*Sample Size and Diversity: The study boasts a relatively large sample size. However, the distribution among categories appears skewed. For instance, there's* ***a discrepancy*** *between confirmed cases of Omicron* ***(297)*** *versus the original strain* ***(73)****, which might* ***influence the comparative outcomes****.*

**Author response:** Thank you very much for constructive comments. Following your advice, we employed a 1:2 propensity score matching to mitigate the impact of confounding factors such as age and gender. Simultaneously, we addressed the issue of sample size imbalance between the two groups. Consequently, we selected 134 Omicron strain-infected individuals and 73 original strain-infected individuals for the analysis of the oropharyngeal microbiota. The results of the reanalysis were similar to the previous results. The oropharyngeal microbial alpha diversity in CCO group was significantly higher than that in CCOS group (p<0.001). The distribution of oropharyngeal microbial community between two groups was significant different. The results of Wilcoxon rank-sum test show that a total of 10 phyla with significant differences in abundance were identified (p<0.05). Between them, 6 phyla such as *Bacteroidota* and *Patescibacteria* were increased in CCO group (p<0.05), while 4 phyla such as *Cyanobacteria* and *Actinobacteriota* were increased in CCOS group (p<0.05).

*Key Findings: The observed microbial imbalance—increased conditional pathogens and decreased acidogenic bacteria—provides a noteworthy insight into potential secondary infections or complications during Omicron infections. Yet, the study could benefit from* ***further elaborating on the direct consequences and underlying mechanisms of these microbial shifts****.*

**Author response:** Thank you very much for constructive comments. Based on your suggestions, we elaborated in the manuscript on the direct consequences and potential mechanisms of microbial alterations. Additionally, we introduced several articles on the oropharyngeal microbiota of COVID-19 patients, offering a more comprehensive supplement to the microbial roles in the disease. The addition of conditional pathogens we introduced in detail *Prevotella* and *Veillonella*. The details could be found in the revised manuscript as follows:

*Prevotella*: The lipopolysaccharide contained in *Prevotella* can activate epithelial cells to produce IL-8, IL-6 and CCL20, thus promoting mucosal Th17 immune response and neutrophil recruitment. Therefore, although *Prevotella* is a resident bacterium, its enrichment may lead to a cytokine storm. And it also closely related to localized infection of ear and nose, inflammatory periodontitis, pulmonary infection, gastrointestinal infection and some autoimmune diseases. Moreover, immunological interactions between *Prevotella* and viruses usually inducing an increase of pro-inflammatory responses. Virulence factors including lipopolysaccharide, fimbriae, hemolysin and adhesin were found in *Prevotella*, which can promote its pathogenicity and survival in the host. These studies show that *prevotella* not only promotes the growth of SARS-CoV-2, but also interacts with certain mediators of disease progression.

Among the reduced acidogenic bacteria, we mainly enumerated *Fusobacterium* and *Alloprevotella*, and we describe in detail their common metabolites short-chain fatty acids, such as butyric acid, propionic acid and so on. Several pieces of evidence show that short-chain fatty acids have the effects of anti-inflammation, antioxidation, inhibiting cancer and promoting the integrity of intestinal epithelial barrier function. Most importantly, butyric acid can not only enhance the antibacterial function of macrophages，but also significantly inhibit the production of proinflammatory cytokines, chemokines and calprotectin by neutrophils, and reduce the release of MPO, ROS and NETs.

*Diagnostic Model: The development of a diagnostic model based on oropharyngeal microbial markers is innovative. However, its real-world application would necessitate validation across* ***varied populations and more extensive sample sizes*** *to ensure generalizability.*

**Author response:** Thank you very much for constructive comments. In order to validate the generalizability of our diagnostic model based on oropharyngeal microbial markers, we included a new validation set comprising 20 Omicron-infected individuals (BA.5.2, the same as our study) and 20 healthy individuals across different age groups. The original discovery set of Omicron-infected individuals had an average age of 29.92, while the average age of confirmed cases of Omicron in the verification phase was 30.25 years old. In the newly included validation set, the average age of Omicron-infected individuals was 53.15 years, and for the healthy cohort, it was 57.65 years. The results indicate that our established diagnostic model accurately distinguishes middle-aged infected individuals from middle-aged healthy individuals, with an AUC value of 98.42% (95% CI 95.14% to 100%, p<0.0001). This suggests a certain level of universality for our diagnostic model.

*Limitations Acknowledgment: While the study acknowledges its limitations—like the inability to* ***stratify disease severity*** *and the* ***lack of animal model validation****—it could have benefited from* ***addressing potential confounding factors****, like the effect of* *concurrent treatments on the microbiome, dietary differences, or geographical variations.*

**Author response:** Thank you very much for constructive comments. Our study lacked validation using animal models. But we conducted an analysis of the oropharyngeal microbiome in mild and moderate patients infected with the Omicron variant, to complement the stratification of disease severity. The PCoA analysis showed that there were significant differences in the composition of oropharyngeal microbial community between the mild patients and moderate patients. At the genus level, the *Streptobacillus*, *Lachnoanaerobaculum* and *Eubacterium Nodatum* of moderate patients was significantly higher than that of mild patients (p<0.05).

Our study controlled for confounding following factors. (1) Regarding **concurrent treatments**, the collection of these samples was in the stage of epidemic control in China, and the government has been carrying out epidemiological investigations of the COVID-19, and once cold-like symptoms appear, they are directly sent to designated hospitals for nucleic acid testing, and over-the-counter drugs such as antipyretics are under control during the epidemic and cannot be purchased by individuals. After the patient is confirmed to be positive for nucleic acid, he will be isolated to the hospital within one day. A throat swab sample is taken immediately after admission. The treatment plan of the patients after admission was based on the "Diagnosis and Treatment Plan for COVID-19 (Trial Ninth Edition)", and antiviral therapy combined with symptomatic supportive care was adopted, and antibiotics and hormones were not used. (2) Regarding **geographical variations**, all samples were collected within Henan Province. (3) Considering **dietary differences**, as patients were treated under centralized isolation, dietary conditions were largely uniform. Moreover, the eating habits of people in the same area are basically similar. Thus, we minimized the impact of confounding factors such as treatment protocols, dietary variations, and geographical differences on the study.

*Causation vs. Correlation: While the study found associations between oropharyngeal microbiomes and COVID-19 Omicron variant infection, it does not necessarily mean* ***the changes in microbiomes caused or were caused by the infection****. Further research is* ***required to establish causality under the light of confounding factors****. Like whether the patients were taking general* ***over the counter medications*** *to mitigate fever and other symptoms etc. before confirmation of covid infection (omicron or not). This may affect the microbiome quite significantly.*

**Author response:** Thank you very much for constructive comments. Our current study results indicate an association between the oropharyngeal microbiome and the COVID-19 Omicron variant. However, this does not necessarily imply that the changes in microbiomes caused or were caused by the infection. SARS-CoV-2 colonization might restrict the survival space of oropharyngeal bacteria, leading to microbial alterations. Conversely, the growth of pathogenic bacteria could potentially promote disease progression through the secretion of substances like LPS. SARS-CoV-2 and bacteria are likely to interact with each other. Throughout our research, we controled the impact of confounding factors such as medications, geographical location, and dietary conditions. Hence, our findings authentically reflect the changes in oropharyngeal microbiota following SARS-CoV-2 infection as accurately as possible.

Regarding the question of whether the patient took oral over-the-counter drugs before infection, the collection of these samples was in the stage of epidemic control in China, and the government has been carrying out epidemiological investigations of the COVID-19, and once cold-like symptoms appear, they are directly sent to designated hospitals for nucleic acid testing, and over-the-counter drugs such as antipyretics are under control during the epidemic and cannot be purchased by individuals. After the patient is confirmed to be positive for nucleic acid, he will be isolated to the hospital within one day. A throat swab sample is taken immediately after admission.

*Conditional Pathogens: The term "conditional pathogens" is vague. It would be beneficial for readers if* ***the specific pathogens were listed****, along with* ***how they might be related to COVID-19 or other infections****.*

**Author response:** Thank you very much for constructive comments. In our revised manuscript, we've introduced the concept of conditional pathogens. The previous manuscript had descriptions that could potentially lead to misinterpretation, so we've refined the descriptions for accuracy. Additionally, we've supplemented the specific pathogens listed with their associations concerning COVID-19 and other infections. The details could be found in the revised manuscript:

First of all, we added its specific concept in time when the conditional pathogen was mentioned for the first time — “microbes that normally exist in the human body cause infection under specific circumstances, such as the decrease of immune function, changes or disorders of settlement sites and so on”.

*Prevotella*: The lipopolysaccharide contained in *Prevotella* can activate epithelial cells to produce IL-8, IL-6 and CCL20, thus promoting mucosal Th17 immune response and neutrophil recruitment. Therefore, although *Prevotella* is a resident bacterium, its enrichment may lead to a cytokine storm. Moreover, immunological interactions between *Prevotella* and viruses usually inducing an increase of pro-inflammatory responses. Virulence factors including lipopolysaccharide, fimbriae, hemolysin and adhesin were found in *Prevotella*, which can promote its pathogenicity and survival in the host. These studies show that *prevotella* not only promotes the growth of SARS-CoV-2, but also interacts with certain mediators of disease progression.

*Veillonella*: Studies have shown that the combination of *Veillonella* and *Streptococcus* can counteract the production of IL-12p70 and enhance the response of IL-8, IL-6, IL-10 and TNF- α, which may be related to the cytokine storm after COVID-19 infection. Ma et al. reported that *Veillonella* was the most prominent biomarker for COVID-19 compared with flu patients or healthy controls, and it also one of the bacteria to predict the severity of COVID-19. Although the types of strains in research are different, it shows that microbiome dysbiosis can promote the inflammatory environment favoring coronavirus invasion and viral replication.

*Potential Confounders: There might be* ***other factors influencing the oropharyngeal microbiome*** *(e.g., diet, antibiotic usage, OTC drugs, other infections etc.). It's essential to consider and account for these* ***potential confounders*** *in the analyses.*

**Author response:** Thank you very much for constructive comments. Our study controlled for confounding following factors. (1) Regarding **antibiotic usage and OTC drugs**, the collection of these samples was in the stage of epidemic control in China, and the government has been carrying out epidemiological investigations of the COVID-19, and once cold-like symptoms appear, they are directly sent to designated hospitals for nucleic acid testing, and over-the-counter drugs such as antipyretics are under control during the epidemic and cannot be purchased by individuals. After the patient is confirmed to be positive for nucleic acid, he will be isolated to the hospital within one day. A throat swab sample is taken immediately after admission. The treatment plan of the patients after admission was based on the "Diagnosis and Treatment Plan for COVID-19 (Trial Ninth Edition)", and antiviral therapy combined with symptomatic supportive care was adopted, and antibiotics and hormones were not used. (2) Regarding **geographical variations**, all samples were collected within Henan Province. (3) Considering **dietary differences**, as patients were treated under centralized isolation, dietary conditions were largely uniform. Moreover, the eating habits of people in the same area are basically similar. (4) We use the PSM to control some common confounding factors as much as possible. For example, the analysis of the characteristics of oropharyngeal microbiomes of patients with two-times vaccine and three-times vaccine, and the analysis of the characteristics of oropharyngeal microbiomes of mild and moderate patients of Omicron variant, we excluding confounding factors such as sex, age, white blood cells, neutrophils, lymphocytes, hemoglobin, platelets, alanine aminotransferase, aspartate aminotransferase, total bilirubin, urea and so on.

Thus, we minimized the impact of confounding factors such as treatment protocols, dietary variations, and geographical differences on the study.

*The phrase "significant difference" is used multiple times, especially in the context of* ***NMDS and PCoA*** *analyses in Section 2.2. It would be helpful to* ***elaborate on what kind of differences were observed****. Were certain bacterial taxa more prevalent in one group than the other? Were there shifts in the overall microbial community composition?*

**Author response:** Thank you very much for constructive comments. In the newly revised manuscript, we explain the "significant difference" in more detail, and the significant difference in the Shannon index mainly refers to the community diversity between the two groups of oropharyngeal microorganisms, and the significant difference between PCoA and NMDS represents a change in the overall microbial community composition. The details could be found in the revised manuscript, as follows:

The results of PCoA and NMDS analysis showed that there was a significant difference in the distribution of oropharyngeal microbial community between CCO group and HC group, the overall microbial community composition changed between the two groups.

*Discussion mentions “NET formation”, which presumably refers to Neutrophil Extracellular Traps. Given the importance of this mechanism in the context of infections, it might be beneficial to* ***explain it briefly****.*

**Author response:** Thank you very much for constructive comments. "NET information" means "neutrophil extracellular traps". We have corrected it and explain it in more details in the manuscript, as follows:

NETs are released by neutrophils, which can kill microorganisms. However, excessive generation of NETs will lead to many negative effects. When neutrophils are exposed to live SARS-CoV-2 virus, they are more likely to form NETs than other neutrophils.

NET production also has been considered as a predictor to assess disease severity and clinical outcomes during infection.

*It is unclear how it may lead to* ***potential clinical implications*** *and* ***applications*** *of the findings. How can these results be used to* ***improve patient care or guide future research****? Worth* ***discussing****.*

**Author response:** Thank you very much for constructive comments. Our research has the following potential application value and significance.

(1) Our study employed a random forest model to establish a non-invasive diagnostic model based on oropharyngeal microbiota in the training set, achieving favorable diagnostic performance in the validation set. Further validation across different age groups demonstrated the **generalizability** of the diagnostic model, while validation using the Omicron subtype XBB.1.5 confirmed its **specificity**. These findings suggest **a novel non-invasive tool for clinically diagnosing Omicron BA.5.2 infections**.

(2) Additionally, this study observed **variations** in certain bacteria **compared to healthy controls**, with some bacteria showing an increase or decrease **in the variant strain.** Correlation analysis revealed associations between oropharyngeal microbiota and specific clinical indicators. **Subgroup analysis** indicated a significant reduction in certain bacteria in patients with **moderate infection compared to mild infection**. Consequently, this study proposes a novel theory that Omicron variant infections may disrupt oropharyngeal microbiota, exacerbating disease severity possibly through the secretion of endotoxins. These discoveries **offer new insights for targeting oropharyngeal microbiota to alleviate disease progression or prevent variant strain infections**.

*The term "packet" in "R packet" (Section 4.5) is typically referred to as "package". Consider revising to "R package.*

**Author response:** Thank you very much for constructive comments. We have revised the“R packed”to “R package”.

*The section 4.5 about the cutoff value for the LDA score might benefit from a rationale: Why were these particular cutoffs chosen?*

**Author response:** Thank you very much for constructive comments. The magnitude of the LDA score can be used to assess the impact of each species' abundance on the effect size of differences. The rationale behind setting the threshold is due to the presence of numerous enriched pathways in the analysis. By setting this threshold, we further screened these pathways to visually highlight the most impactful pathways within the groups in the graphical representation. The results for all the pathways with differences are presented in the supplementary table.

*The term* ***Oropharynx*** *should be used in the title and elsewhere* ***instead of oropharyngeal****, wherever relevant. The manuscript requires a comprehensive review for* ***linguistic accuracy and clarity*** *in its expression.*

**Author response:** Thank you very much for constructive comments. We've changed oropharyngeal to oropharynx in the title and elsewhere. At the same time, we have conducted a comprehensive review of the accuracy and clarity of the language expression of the whole article, in order to express the content more clearly and accurately.

**Part II – Major Issues: Key Experiments Required for Acceptance**

*Analysis of the data under the light of confounding factors and incorporation of more healthy controls for better comparisons*

**Author response:** Thank you very much for constructive comments. Our study controlled for confounding following factors. (1) Regarding **antibiotic usage and OTC drugs**, the collection of these samples was in the stage of epidemic control in China, and the government has been carrying out epidemiological investigations of the COVID-19, and once cold-like symptoms appear, they are directly sent to designated hospitals for nucleic acid testing, and over-the-counter drugs such as antipyretics are under control during the epidemic and cannot be purchased by individuals. After the patient is confirmed to be positive for nucleic acid, he will be isolated to the hospital within one day. A throat swab sample is taken immediately after admission. The treatment plan of the patients after admission was based on the "Diagnosis and Treatment Plan for COVID-19 (Trial Ninth Edition)", and antiviral therapy combined with symptomatic supportive care was adopted, and antibiotics and hormones were not used. (2) Regarding **geographical variations**, all samples were collected within Henan Province. (3) Considering **dietary differences**, as patients were treated under centralized isolation, dietary conditions were largely uniform. Moreover, the eating habits of people in the same area are basically similar.

We included an additional 20 healthy controls in the middle-aged to elderly group to expand our sample size. Through ROC curve analysis, we confirmed the strong diagnostic efficacy of our microbial markers across different age brackets.

*Association of the findings with disease severity or response to treatment. Whether the patients were vaccinated and their response/time of vaccination should be considered.*

**Author response:** Thank you very much for constructive comments.

(1) We utilized propensity score matching to control confounding factors such as age, gender, white blood cells, neutrophils, lymphocytes, hemoglobin, alanine aminotransferase, aspartate aminotransferase, albumin, total bilirubin, urea, and number of vaccine injections. This allowed us to conduct a specific analysis involving **57 mild infection cases and 19 moderate infection cases**. We identified distinctive microbial variations between these two groups, unveiling novel microbial targets for mitigating disease progression through targeted microbiome intervention. The detailed results could be found in the revised manuscript as follows:

The results of Shannon index showed that there was no significant difference in microbial α-diversity between moderate and mild patients. The PCoA analysis showed that there were significant differences in the composition of pharyngeal microbial community between the two groups. Further analysis of the differences in the expression of pharyngeal microbial between mild and moderate patients at different classification levels showed that there was no significant difference between the two groups of microbial at the phylum level (p>0.05). At the genus level, the *Streptobacillus*, *Lachnoanaerobaculum* and *Eubacterium Nodatum* of moderate patients was significantly higher than that of mild patients (p<0.05). In the Kyoto Encyclopedia of Genome and Genome (KEGG) database, we found that there was no significant difference in metabolic pathways between mild and moderate patients.

(2) The diagnostic criteria and treatment protocols for patients enrolled in this study were based on the " Diagnosis and Treatment Plan for COVID-19 (Trial Ninth Edition)". Our approach included a combination of antiviral therapy and symptomatic supportive treatment, ensuring uniformity in treatment strategies among participants. Consequently, we did not conduct an analysis focusing on **divergent treatment approaches**.

(3) In order to further study whether **the efficacy of the vaccine is related to the imbalance of oropharyngeal microecology**, we adopted the PSM method by excluding confounding factors such as sex, age, severity of disease, white blood cells, neutrophils, lymphocytes, hemoglobin, platelets, alanine aminotransferase, aspartate aminotransferase, total bilirubin, urea and so on. 60 patients who received two doses of COVID-19 vaccine (CCO2) and 20 patients who received three doses of COVID-19 vaccine (CCO3) were selected to analyze the differential expression of bacterial species between the two groups by Willcoxon rank sum test. Our results show that there is no significant difference between the two groups at the phylum level (p>0.05). At the genus level, we find enrichment of *Granulicatella*, *Lactobacillales* and *Corynebacterium* in CCO2 compared with CCO3 (p<0.05).

*More clarity is needed on the 'diagnostic model of oropharyngeal microbial markers' perhaps comparing with the disease outcomes and response to drugs/vaccines. Clinical potential seems to be there but not clear.*

**Author response:** Thank you very much for constructive comments.

(1) The project had no mortality outcomes; all patients recovered and were discharged. Following your advice, we conducted a subgroup analysis based on disease severity. Fifty-seven cases of mild infection and nineteen cases of moderate infection were included. Differential analysis revealed key bacteria associated with disease severity, including *Streptobacillus*, *Lachnoanaerobaculum*, and *Eubacterium nodatum*. This microbial perspective offers novel targets to attenuate disease progression and improve clinical outcomes.

(2) The diagnostic criteria and treatment protocols for patients enrolled in this study were based on the " Diagnosis and Treatment Plan for COVID-19 (Trial Ninth Edition)". The patients were treated with antiviral therapy combined with symptomatic supportive care, and the regimen was consistent, and all patients were discharged after treatment. However, **the number of vaccinations varied among the participants**, and a subgroup analysis of the number of vaccinations as recommended by you found that *Granulicatella*, *Lactobacillales* and *Corynebacterium* were significantly higher in the group of patients who received 2 times of the vaccine. The effect of vaccination on oropharyngeal microbiota was analyzed, and a new auxiliary target targeting microbiota was provided for enhancing the protective efficacy of vaccines after infection.

(3.1) This study elucidates the unique oropharyngeal microbiota features of the Omicron variant, identifying bacteria significantly different from those in healthy individuals. It reports the gradual changes in bacteria with disease recovery, potentially linked to disease progression and post-COVID sequelae. Employing PSM to eliminate confounding factors, a specific analysis was conducted on 57 cases of mild infection and 19 cases of moderate infection, identifying key bacteria influencing disease severity. This offers novel targets in mitigating disease progression through microbiome modulation.

(3.2) We developed a non-invasive diagnostic model for COVID-19 through training and validation sets. To demonstrate the model's applicability, we included a cross-age validation cohort, affirming its robust diagnostic performance across different age groups. Further validation involving the Omicron subvariant XBB.1.5 confirmed the accuracy of our diagnostic markers in distinguishing between the BA.5.2 and XBB.1.5 strains, indicating the microbial markers' specificity to the Omicron variant and clinical adaptability.

(3.3) This project pioneers the analysis of the microbiota differences between the original strain and the Omicron variant, uncovering alterations in the microbiota after infection with the Omicron BA.5.2 variant compared to the original strain.

**Part III – Minor Issues: Editorial and Data Presentation Modifications**

*The legends accompanying the figures could benefit from further elaboration, including the addition of specific experimental details and the statistical tests employed. Additionally, the clarity and presentation of the figures themselves should be enhanced, as their current quality appears to be compromised.*

**Author response:** Thank you very much for constructive comments. We have further elaborated the figure legends including specific experimental details and the statistical tests employed. Moreover, we have improved the clarity of the figure. The details could be found in the revised manuscript.

Please contact us if there are any further questions.

Thank you very much for your consideration.

Best Regards

Sincerely yours,

Zhigang Ren, PhD.,M.D.

Department of Infectious Diseases, the First Affiliated Hospital of Zhengzhou

University, Zhengzhou, 450052, China.

E-mail: [fccrenzg@zzu.edu.cn](mailto:fccrenzg@zzu.edu.cn)

December 28, 2023
