# Supplementary material for: Dynamic changes of Bacterial Microbiomes in Oropharynx during Infection and Recovery of COVID-19 Omicron Variant
Source: PLoS Pathog. 2024 Apr 3;20(4):e1012075. doi: 10.1371/journal.ppat.1012075 (PMC10990182; doi:10.1371/journal.ppat.1012075)
Supplement: Attachment — Submitted filename: renamed_f9ec3.docx [file ppat.1012075.s048.docx]

February 21, 2024

Ashley L. St. John

Academic Editor

PLOS Pathogens

Ana Fernandez-Sesma

Section Editor

PLOS Pathogens

Michael Malim

Editor-in-Chief

PLOS Pathogens

RE: Dynamic changes of Bacterial Microbiomes in Oropharynx during Infection and Recovery of COVID-19 Omicron Variant (PPATHOGENS-D-23-01358R1)

Dear Editor:

Thank you very much for your help and great contribution on our manuscript entitled "Dynamic changes of Bacterial Microbiomes in Oropharynx during Infection and Recovery of COVID-19 Omicron Variant" (PPATHOGENS-D-23-01358R1).

We have made a detailed response based on the latest comments from editors and reviewers. The details can be found as follows. We have revised the manuscript, and carefully proofread the whole manuscript. The revised manuscript has been edited and polished for proper English language, grammar, punctuation, spelling, and overall style. The reviewer comments are laid out below in blue text and specific concerns have been numbered. Our response is given in black text.

We resubmit the revised manuscript, including figures and the point-to-point response letter.

**COMMENTS TO Reviewers:**

**Part I – Summary**

**Reviewer #1**:

*The authors have done a good job responding to the reviewer comments.*

**Author response:** We are honored to have your approval, and if you have any new ideas in the next adjustment, please feel free to let us know.

**Reviewer #2**:

*Authors have done a good job at comparative analysis in the revised manuscript and have answered all the questions raised during the review.*

**Author response:** It's an honor to have your approval. In the follow-up review process, if you have any questions, please feel free to let us know and look forward to communicating with you.

**Part II – Major Issues: Key Experiments Required for Acceptance**

**Reviewer #1**:

*(No Response)*

**Reviewer #2**:

*Authors have answered all my questions with satisfactory explanation and evidences through analysis.*

**Author response:** Thank you for your valuable comments on our research and for your recognition of our work.

**Part III – Minor Issues: Editorial and Data Presentation Modifications**

**Reviewer #1**:

*Line 46 restructure sentence to something like & quot; Oropharyngeal microbiomes play a significant role in the susceptibility and severity of COVID-19, yet the role these microbiomes play for the development of COVID-19 omicron variant have not been reported.*

*line 53 define the best*

*line 54 define OTU*

**Author response:** Thank you very much for constructive comments. In the manuscript, we have changed the sentence “The role of oropharyngeal microbiomes in the susceptibility and severity of disease cannot be ignored, but the characteristics of oropharyngeal microbiomes of COVID-19 Omicron variant have not been reported” of line 46 into “Oropharyngeal microbiomes play a significant role in the susceptibility and severity of COVID-19, yet the role of these microbiomes play for the development of COVID-19 Omicron variant have not been reported”.

In order to avoid ambiguity, we changed line 53 related to “best” of sentence “Based on the six best oropharyngeal OTUs” into “Based on six optimal oropharyngeal operational taxonomy units (OTUs)”.

For the accuracy of the manuscript, we changed the OTUs in line 54 to the full name “operational taxonomy units”.

**Reviewer #2**:

*(No Response)*

Please contact us if there are any further questions.

Thank you very much for your consideration.

Best Regards

Sincerely yours,

Zhigang Ren, PhD.,M.D.

Department of Infectious Diseases, the First Affiliated Hospital of Zhengzhou

University, Zhengzhou, 450052, China.

E-mail: [fccrenzg@zzu.edu.cn](mailto:fccrenzg@zzu.edu.cn)

February 21, 2024
